# A Protein Aggregation Based Test for Screening of the Agents Affecting Thermostability of Proteins

**DOI:** 10.1371/journal.pone.0022154

**Published:** 2011-07-08

**Authors:** Tatyana Eronina, Vera Borzova, Olga Maloletkina, Sergey Kleymenov, Regina Asryants, Kira Markossian, Boris Kurganov

**Affiliations:** 1 Bach Institute of Biochemistry, Russian Academy of Sciences, Moscow, Russia; 2 Kol'tsov Institute of Developmental Biology, Russian Academy of Sciences, Moscow, Russia; 3 Belozersky Institute of Physico-Chemical Biology, Moscow State University, Moscow, Russia; Aston University, United Kingdom

## Abstract

To search for agents affecting thermal stability of proteins, a test based on the registration of protein aggregation in the regime of heating with a constant rate was used. The initial parts of the dependences of the light scattering intensity (*I*) on temperature (*T*) were analyzed using the following empiric equation: *I* = *K*_agg_(*T*−*T*_0_)^2^, where *K*_agg_ is the parameter characterizing the initial rate of aggregation and *T*_0_ is a temperature at which the initial increase in the light scattering intensity is registered. The aggregation data are interpreted in the frame of the model assuming the formation of the start aggregates at the initial stages of the aggregation process. Parameter *T*_0_ corresponds to the moment of the origination of the start aggregates. The applicability of the proposed approach was demonstrated on the examples of thermal aggregation of glycogen phosphorylase *b* from rabbit skeletal muscles and bovine liver glutamate dehydrogenase studied in the presence of agents of different chemical nature. The elaborated approach to the study of protein aggregation may be used for rapid identification of small molecules that interact with protein targets.

## Introduction

Senisterra and coworkers [1], [2] elaborated a high-throughput light-scattering-based method for screening of ligands specifically interacting with protein targets. Thermal protein denaturation is used to characterize the binding of ligands to their target protein. This method is based on the assumption that the proteins under study irreversibly denaturate and form aggregates during thermal denaturation. Light scattering as a measure of protein aggregation is a very sensitive technique. Protein aggregation is studied in the regime of heating with a constant rate. The dependence of the light scattering intensity on temperature has a sigmoid shape. At rather high temperatures the light scattering intensity (*I*) reaches a limiting value (*I*_lim_). To characterize thermostability of a protein, Senisterra and coworkers used the temperature (*T*_agg_) corresponding to the middle point of the transition, i.e., a temperature at which *I* = *I*_lim_/2. Parameters *I*_lim_ and *T*_agg_ are determined by fitting of the experimental dependence of *I* on temperature with the following empiric equation, analogous to the Boltzmann equation:
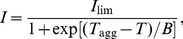
(1)where *B* is a constant.

According to the idea advanced by Senisterra and coworkers a change in the *T*_agg_ value in the presence of a ligand characterizes the effect of the latter on protein thermostability. To substantiate this conclusion, the authors additionally used differential scanning calorimetry (DSC), which is a source of direct information on the protein resistance to high-temperature exposure. The authors also constructed a plot demonstrating the existence of a correlation between the *T*_agg_ value and the position of the maximum on the DSC profiles (*T*_m_).

It is evident that the accuracy of determining parameter *T*_agg_ is connected with the reliability of the estimation of parameter *I*_lim_. When trying to estimate parameter *I*_lim_ we should take into account that the “true” limiting level of the light scattering intensity may not be reached because of precipitation of the large-sized aggregates formed at high temperatures. Such a precipitation results in the decrease in the light scattering intensity, and the real experimental dependence of *I* on temperature looks like a curve passing through a maximum. The maximum value of the light scattering intensity may be lower than the *I*_lim_ value calculated from Eq. (1). Besides, the correlation between the increment of the light scattering intensity and the degree of protein denaturation should be controlled not only by checking the correlation between parameters *T*_agg_ and *T*_m_, but by stricter analysis of the relationship between turbidimetric data and calorimetric data, supplying direct information on the degree of protein denaturation.

To avoid the uncertainty in the estimation of parameter *I*_lim_, in the present work we proposed new parameters, which characterize the rate of aggregation. To determine these parameters, there is no need for the full dependences of the light scattering intensity on temperature, since the proposed parameters (the initial temperature of aggregation and the parameter characterizing the rate of change in the light scattering intensity with temperature) are calculated from the initial parts of the dependences of *I* on temperature. The use of these parameters allows us to obtain the quantitative characteristics of the effect of the agents to be tested on the rate of aggregation. Since the initial stage of thermal aggregation of the proteins is the stage of denaturation, the ligands under study involve agents affecting both the stage of denaturation and the stage of aggregation. To demonstrate the applicability of the proposed approach, thermal aggregation of glycogen phosphorylase *b* (Ph*b*; EC 2.4.1.1) from rabbit skeletal muscles and bovine liver glutamate dehydrogenase (GDH; EC 1.4.1.3) were used as examples.

### Mathematical description of the initial parts of the dependences of the light scattering intensity on temperature

As a preliminary step we discuss the kinetics of thermal aggregation of the proteins registered at a fixed temperature. To analyze the initial parts of the dependences of the light scattering intensity (*I*) on time, the following empiric equation was proposed [3]:
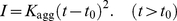
(2)In this equation *K*_agg_ is a constant with the dimension of (counts/s)⋅min^−2^ and *t*_0_ is the duration of the lag period (*t*_0_ is a point in time at which the light scattering intensity begins to increase). The applicability of Eq. (2) was demonstrated for thermal aggregation of Ph*b* [3]–[5], glyceraldehyde-3-phosphate dehydrogenase (GAPDH; EC 1.2.1.12) [6] and creatine kinase (CK; EC 2.7.3.2) from rabbit skeletal muscles [7]. Parameter *K*_agg_ characterizes the initial rate of aggregation, and the construction of a dependence of parameter *K*_agg_ on the initial protein concentration [P]_0_ allows calculating the order of aggregation with respect to the protein (*n*) (*K*_agg_ = const⋅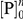). The dependence of parameter *K*_agg_ on the Ph*b* concentration constructed on the basis of kinetic data for Ph*b* aggregation at 53°C (pH 6.8) was linear [3], and consequently *n* = 1. This is true when the stage of unfolding of a protein molecule proceeds with a substantially lower rate than the following stages of aggregation of the unfolded protein molecules [8], [9]. In other words, the rate-limiting stage of the overall process of aggregation is the monomolecular stage of protein unfolding. According to the data presented by Kurganov et al. [10] the kinetic scheme of thermal denaturation of Ph*b* involves a stage of the conformational change of the enzyme dimeric molecule followed by dissociation of the dimer into monomers and denaturation of labile monomeric forms.

The kinetics of thermal aggregation of GDH at various concentrations of the protein was studied by Sabbaghian et al. [11] (50°C; pH 8.0). The order of aggregation with respect to the protein calculated on the basis of these kinetic data is close to unity: *n* = 0.8±0.1.

The analysis of the data on thermal aggregation of β_L_-crystallin from bovine lens at 60°C (pH 6.8) obtained in [12] shows that parameter *n* is close to 2 (*n* = 1.99±0.07). It is generally accepted that the initial stage of aggregation is the stage of nucleation [3], [8], [9], [13]–[19]. The fact that the value of parameter *n* for thermal aggregation of β_L_-crystallin exceeds unity is indicative of the involvement of several molecules of denatured β_L_-crystallin in the nucleation process.

Consider a situation when Eq. (2) may be strictly fulfilled. The theoretical analysis carried by Ferrone [16] shows that for the nucleation-dependent aggregation the accumulation of monomers incorporated in the aggregate (Δ) proceeds according to the time-squared law:
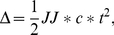
(3)where *J* is the elongation rate of aggregates, J* is the elongation rate of the nucleus and c* is the concentration of the nuclei. Such an early kinetics of aggregation was demonstrated experimentally, for example, for aggregation of polyglutamine peptides [20], aggregation of a slow-folding mutant of a β-clam protein, cellular retinoic acid-binding protein (P39A CRABP I) [21], and aggregation of protein L [22].

When studying the kinetics of thermal aggregation of some proteins by dynamic light scattering (DLS) we have shown that the hydrodynamic radius (*R*_h_) of the initially registered aggregates (the start aggregates) amounts to tens of nanometers [23]. This means that a start aggregate contains hundreds of denatured protein molecules. We could not detect intermediate states between the original forms of the proteins and the start aggregates. It should be noted that rather low temperatures were selected for the study of protein aggregation in this investigation (37°C for Ph*b* and 45°C for GAPDH). The nuclei formed at the initial stages of aggregation involve several monomers. Therefore one can assume that there is no accumulation of nuclei in the system, and coalescence of the formed nuclei into larger structures, namely start aggregates, takes place. The initial increase in the light scattering intensity is connected with accumulation of the start aggregates, the size of the latter remaining unchanged (the hydrodynamic radius of the start aggregates was designated by *R*_h,0_). It is evident that in the time interval where the *R*_h_ value is constant (*R*_h_ = *R*_h,0_; see [23]) the light scattering intensity is strictly proportional to the amount of the aggregated protein. Thus, in this case Eq. (2) may be used for the quantitative description of the early kinetics of the aggregation process, and parameter *K*_agg_ is a true measure of the initial rate of aggregation. When protein aggregation is studied at elevated temperatures, the time interval corresponding to the accumulation of the start aggregates is greatly reduced, and the registered increase in the hydrodynamic radius of the protein aggregates at *t*>*t*_0_ is due to the attachment of individual denatured protein molecules to the start aggregates or the sticking of the start aggregates. This results in the additional increment in the light scattering intensity apart from the main contribution accounted for the formation of the start aggregates. Eq. (2) should be treated as an empiric equation where parameter *K*_agg_ is a rough characteristic of the initial aggregation rate.

When studying the dependences of the hydrodynamic radius of protein aggregates on temperature obtained in the experiments with heating of protein solutions with a constant rate, we observed that the character of these dependences is identical to that for the dependences of the hydrodynamic radius of protein aggregates on time obtained at a fixed temperature [24]–[26]. Therefore we propose to use the following equation analogous to Eq. (2) for analysis of the initial parts of the dependences of the light scattering intensity on temperature obtained in the regime of heating with a constant rate:
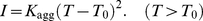
(4)In this equation *T*_0_ is the initial temperature of aggregation, i.e., the temperature at which the light scattering intensity begins to increase, and *K*_agg_ is a constant with the dimension of (counts/s)⋅°C^−2^ Parameter *T*_0_ indicates the moment of origination of the start aggregates, whereas parameter *K*_agg_ characterizes the rate of aggregation. It was of interest to check the applicability of Eq. (4) for the analysis of protein aggregation in the regime of heating with a constant rate, and to clarify how parameters *T*_0_ and *K*_agg_ change with variation in the protein concentration.

## Materials and Methods

Hepes, glucose 1–phosphate, AMP, trimethylamine N-oxide (TMAO), creatine kinase (CK) from rabbit skeletal muscles, bovine liver GDH, NAD, NADH, ADP, L-glutamate, L-leucine and α-crystallin were purchased from “Sigma” (USA). The concentrations of CK, GDH and α-crystallin were determined from the absorbance at 280 nm using the extinction coefficients 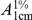 of 8.8 [27], 9.7 [11] and 8.5 [28], respectively. NaCl was purchased from “Reakhim” (Russia), 2-hydroxypropyl-β-cyclodextrin (degree of substitution 3±1) (HP-β-CD) was obtained from CycloLab LTD (Hungary).

### Isolation of Phb

Ph*b* was isolated from rabbit skeletal muscles as described in [29]. Ph*b* concentration was determined spectrophotometrically at 280 nm using the extinction coefficient 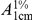 of 13.2 [30].

### Isolation of GAPDH

GAPDH was isolated from rabbit skeletal muscles as described by Scopes and Stoter [31] with an additional purification step using gel-filtration on Sephadex G-100. GAPDH concentration was determined spectrophotometrically at 280 nm using the extinction coefficient 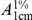 of 10.6 [32].

### Differential Scanning Calorimetry

Thermal denaturation of Ph*b* was investigated by DSC using the adiabatic scanning microcalorimeter DASM–4M (Institute of Biological Instruments, Russian Academy of Sciences, Pushchino, Russia) with 0.47 ml capillary platinum cells. All measurements were carried out at the rate of heating by 1°C/min using the temperature range from 20 to 85°C and constant pressure of 2.2 atm. The dependences of the heat power on temperature were calculated using the program Origin software (MicroCal, Inc., USA).

The capillary construction of calorimetric cells prevents the artifacts caused by protein precipitation, which are often observed in batch calorimetric cells as exothermic peaks. In the capillary cells large-sized aggregated particles of protein are unable to settle and stir the solution, so no extra thermal production can arise [33].

### Light Scattering Intensity Measurements

DLS measurements were performed on a commercial instrument Photocor Complex (Photocor Instruments Inc., USA; www.photocor.com). An He-Ne laser (Coherent, USA, Model 31-2082, 632.8 nm, 10 mW) was used as the light source. The temperature of sample cell was controlled by the proportional integral derivative (PID) temperature controller to within ±0.1°C. To carry on the light scattering intensity measurements in the regime of heating with a constant rate, a fast thermostat with the uniform distributed heater has been developed. The special design of this compact unit allows simply replacing the standard sample cell holder of the main thermostat of the Photocor Complex setup by the fast thermostat. Fast platinum thermometers with time constant of 1 s have been used both for temperature control and for real-time monitoring of temperature directly in the sample cell. The fast thermostat is fully controlled with the existing PID controller through the macro procedure of the Photocor program. Temperature scanning rate can be assigned from 10°C/min to any slower value.

A quasi-cross correlation photon counting system with two photomultiplier tubes was used to increase the accuracy of particle sizing in the range from 1.0 nm to 5.0 µm. DLS data have been accumulated and analyzed with a multifunctional real-time correlator. DynaLS software (Alango, Israel) was used for polydispersity analysis of DLS data. The diffusion coefficient *D* of the particles is directly related to the decay rate τ_c_ of the time-dependent correlation function for the light-scattering intensity fluctuations: *D* = 1/2τ_c_ *k* ^2^. In this equation *k* is the wave number of the scattered light, *k* = (4π*n*/λ)sin(θ/2), where *n* is the refractive index of the solvent, λ is the wavelength of the incident light in vacuum and θ is the scattering angle. The mean hydrodynamic radius of the particles, *R*_h_, can then be calculated according to the Stokes-Einstein equation: *D* = *k_B_T*/6πη*R*_h_, where *k*_B_ is Boltzmann's constant, *T* is the absolute temperature and η is the shear viscosity of the solvent.

All solutions for light scattering experiments were prepared using deionized water obtained with Easy-Pure II RF system (Barnstead). The buffer was placed in a cylindrical cell with a diameter of 6.3 mm and incubated for 5 min at 25°C. Cells with stoppers were used for incubation at high temperature to avoid evaporation. The aggregation process was initiated by the addition of an aliquot of protein to a final volume of 0.5 ml. When studying the kinetics of aggregation of proteins, the scattering light was collected at 90° scattering angle.

Dimensions of the fast thermostat used for heating of the protein solution were the following: 15 mm (diameter)×40 mm (height). Temperature gradients in such a thermostat are less than 0.1 degree. Notice that large temperature gradients in the sample cell would result in a developed convection of fluid. The correlation function of light, scattered on the convective flow, is quite different from the exponential correlation function in case of light scattering on diffusing particles. That is why we can reliably estimate the appearance of temperature gradients by analyzing measured correlation functions.

### Calculations

OriginPro 8.0 SR0 software (OriginLab Corporation, USA) and Scientist software (MicroMath, Inc., USA) were used for the calculations. To characterize the degree of agreement between the experimental data and calculated values, we used the coefficient of determination *R* ^2^ (without considering the statistical weight of the measurement results) [34]:
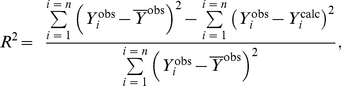
(5)where 
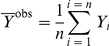
 is the average of the experimental data (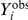), *Y* ^calc^ is the theoretically calculated value of the function *Y* and *n* is the number of measurements.

## Results and Discussion

### Turbidimetric Registration of Phb Aggregation

**Figure 1:**
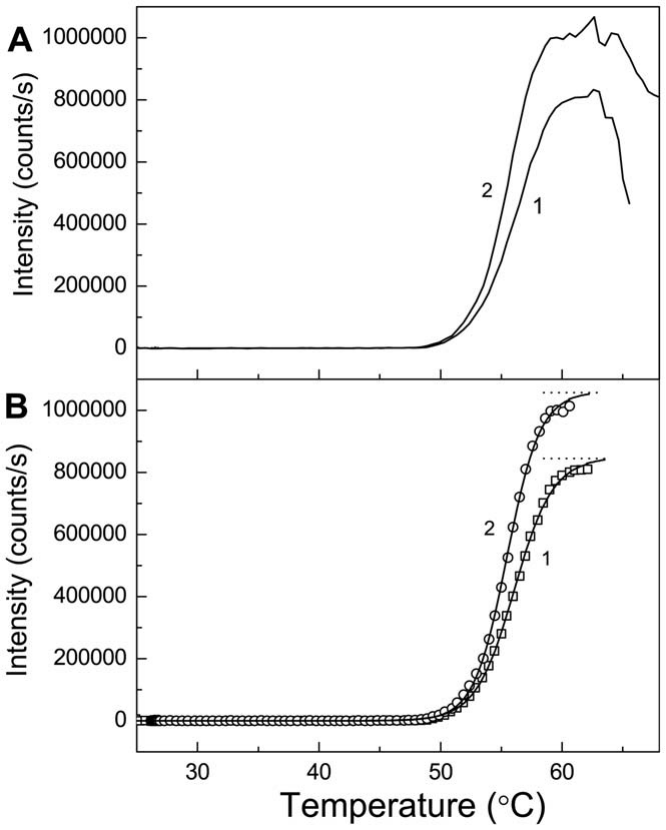
The dependences of the light scattering intensity at 632.8 nm on temperature for thermal aggregation of Ph*b* registered in the regime of heating with a constant rate (1°C/min). Conditions: 0.08 M Hepes-buffer, pH 6.8, containing 0.1 M NaCl. (**A**) The original experimental dependences. The Ph*b* concentrations were as follows: (1) 0.95 and (2) 1.9 mg/ml. (**B**) The results of fitting the experimental curves to Eq. (1). Points are the experimental data. The solid curves are calculated from Eq. (1). The dotted horizontal lines correspond to the limiting values of the light scattering intensity (*I*_lim_).

Thermal aggregation of Ph*b* was studied in the regime wherein temperature was elevated with a constant rate (1°C/min). Fig. 1A shows the dependences of the light scattering intensity on temperature for aggregation of Ph*b* at concentrations of 0.95 and 1.9 mg/ml (curves 1 and 2, respectively). When the temperature is elevated, the light scattering intensity reaches the limiting value. The decrease in the light scattering intensity at rather high temperatures is due to precipitation of the large-sized aggregates. Fig. 1B demonstrates the results of fitting Eq. (1) to the experimental data. The following values of parameters were established: *I*_lim_ = (0.84±0.01)⋅10^6^ counts/s, *T*_agg_ = 56.1±0.1, *B* = 1.57±0.02 (*R* ^2^ = 0.999) at the Ph*b* concentration of 0.95 mg/ml and *I*_lim_ = (1.05±0.01)⋅10^6^ counts/s, *T*_agg_ = 55.5±0.1, *B* = 1.34±0.02 (*R* ^2^ = 0.999) at the Ph*b* concentration of 1.9 mg/ml.

### Denaturation of Ph*b* Registered by DSC and Comparison of the Turbidimetric and Calorimetric Data

**Figure 2:**
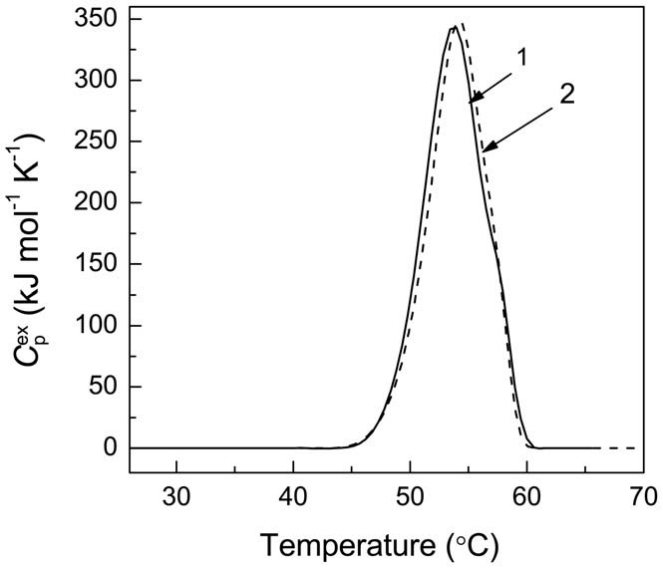
Thermal denaturation of Ph*b* (0.08 M Hepes-buffer, pH 6.8, containing 0.1 M NaCl). The dependences of the excess heat capacity (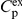) on temperature, obtained at the following concentrations of Ph*b*: (1) 0.95 and (2) 1.9 mg/ml. 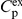 was calculated per dimer of Ph*b* with the molecular mass of 194.8 kDa. The heating rate was 1°C/min.

When studying thermal aggregation of Ph*b*, we should take into account that the initial stage of the general process of aggregation is the stage of protein denaturation. To characterize Ph*b* denaturation, we used DSC. Fig. 2 shows DSC profiles obtained at the Ph*b* concentrations of 0.95 and 1.9 mg/ml (curves 1 and 2, respectively). The heating rate was 1°C/min. The increase in the Ph*b* concentration from 0.95 to 1.9 mg/ml results in the displacement of the maximum on the DSC profiles (*T*_m_) from 53.7±0.1 to 54.2±0.1°C. This change in the shape of the DSC profile with variation of the Ph*b* concentration agrees with the dissociative mechanism of thermal denaturation of Ph*b* [10].

**Figure 3:**
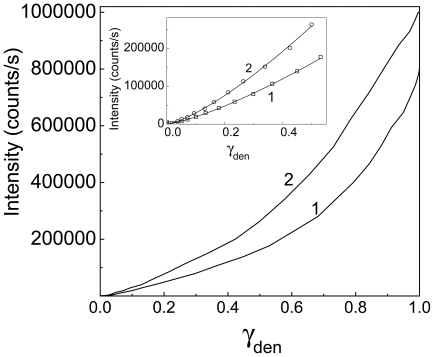
The relationships between the increment of the light scattering intensity accompanying Ph*b* aggregation and the portion of the denatured Ph*b* (γ_den_) calculated from the DSC data. Ph*b* concentrations were as follows: (1) 0.95 and (2) 1.9 mg/ml. The inset shows the initial parts of the curves. Points are the experimental data. The solid curves are calculated from Eq. (6).

When a definite temperature has been achieved, the portion of the protein denatured by this temperature may be calculated from the area under the part of the DSC profile limited at this temperature (*Q*). If we know the area under the whole 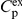 versus temperature profile (*Q*_total_), the portion of denatured protein (γ_den_) is equal to the *Q/Q*_total_ ratio. Fig. 3 shows the relationships between the light scattering intensity registered in the course of Ph*b* aggregation and the γ_den_ value at two concentrations of Ph*b*. It can be seen that the increase in the light scattering intensity (*I*) is not a linear function of γ_den_. Thus, in the case of thermal aggregation of Ph*b* the increment of the light scattering intensity cannot be considered as a direct measure of the degree of protein denaturation. It should be noted that in some cases a linear proportionality between the increase in the light scattering intensity and the degree of protein denaturation is really fulfilled. This, for example, was demonstrated by us for thermal aggregation of GAPDH [35].

The analysis of the relationship between the increment of the light scattering intensity (*I*) and the portion of the denatured Ph*b* presented in Fig. 3 showed that at γ_den_<0.5 the *I* value was a power function of γ_den_:
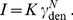
(6)The following values of parameters *K* and *N* were found: *K* = (4.0±0.1)⋅10^5^ counts/s and *N* = 1.32±0.02 (*R* ^2^ = 0.999) at the Ph*b* concentration of 0.95 mg/ml and *K* = (6.6±0.1)⋅10^5^ counts/s and *N* = 1.34±0.02 (*R* ^2^ = 0.999) at the Ph*b* concentration of 1.9 mg/ml.

When comparing calorimetric and turbidimetric data obtained in the regime of heating with a constant rate, we should take into account the following features of the temperature dependences of excess heat capacity and the light scattering intensity. There is no threshold temperature on the DSC profile. The dependence of excess heat capacity on temperature asymptotically approaches zero level with decreasing temperature. The lower the temperature, the lower is the amount of the denatured protein accumulated to the moment when the given temperature has been achieved. On the contrary, an initial increase in the light scattering intensity is registered at definite temperature designated as *T*_0_. According to the mechanism of thermal aggregation of proteins proposed in our works [12], [23], [35]–[37], the initial increase in the light scattering intensity corresponds to the moment of the origination of the start aggregates. The formation of the start aggregates proceeds on the all-or-none principle. The intermediate states between native enzyme forms (or denatured protein molecules) and start aggregates have not been detected.

### Analysis of the Initial Parts of the Dependences of the Light Scattering Intensity on Temperature

**Figure 4:**
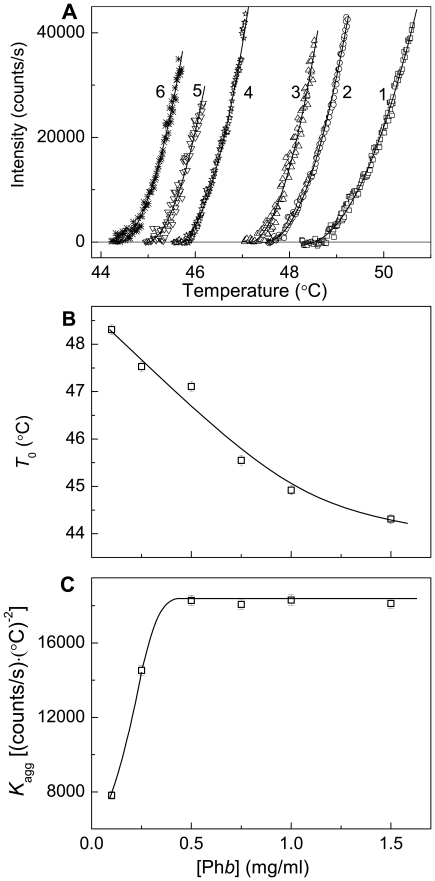
Analysis of the kinetics of Ph*b* aggregation registered in the regime of heating at the rate of 1°C/min. (**A**) The initial parts of the dependences of the light scattering intensity on temperature obtained at the following concentrations of Ph*b*: (1) 0.1, (2) 0.25, (3) 0.5, (4) 0.75, (5) 1.0 and (6) 1.5 mg/ml. Points are the experimental data. The solid curves were calculated from Eq. (4). (**B** and **C**) The dependences of parameters *T*_0_ and *K*_agg_ calculated from Eq. (4) on the Ph*b* concentration, respectively.

In the present work the focus is concentrated on the initial parts of the dependences of the light scattering intensity on temperature. Fig. 4A shows the influence of the protein concentration on the initial parts of the kinetic curves of aggregation of Ph*b* heated with a constant rate of 1°C/min. The Ph*b* concentration was varied in the interval from 0.1 to 1.5 mg/ml. The experimental curves are satisfactorily described by the Eq. (4). The *R* ^2^ values lie in the range from 0.995 at [Ph*b*] = 0.1 mg/ml to 0.990 at [Ph*b*] = 1.5 mg/ml. The calculated values of parameters *T*_0_ and *K*_agg_ are represented in Fig. 4B and C as a function of the Ph*b* concentration. Parameter *T*_0_, characterizing the temperature at which the light scattering intensity begins to increase, decreases monotonously with increasing the concentration of Ph*b* (Fig. 4B).

When interpreting the dependence of parameter *K*_agg_ characterizing the rate of aggregation on the Ph*b* concentration, we should be aware that this dependence can not be used for estimation of the order of aggregation with respect to the protein because the *K*_agg_ values on the *K*_agg_ versus the Ph*b* concentration plot correspond, in fact, to different temperatures. It is of interest that at the Ph*b* concentration higher than 0.5 mg/ml parameter *K*_agg_ is a constant value, which is independent of the protein concentration (Fig. 4C). When the Ph*b* concentration is lower than 0.5 mg/ml, decreasing the protein concentration results in the decrease in the *K*_agg_ value.

**Figure 5 pone-0022154-g005:**
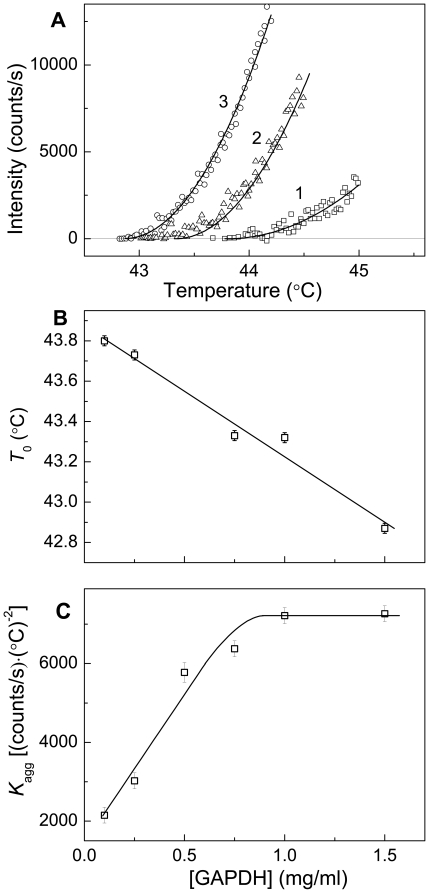
Analysis of the kinetics of GAPDH aggregation registered in the regime of heating at the rate of 1°C/min (10 mM Na-phosphate buffer, pH 7.5, containing 0.1 M NaCl). (**A**) The initial parts of the dependences of the light scattering intensity on temperature obtained at the following concentrations of GAPDH: (1) 0.1, (2) 0.75 and (3) 1.5 mg/ml. Points are the experimental data. The solid curves were calculated from Eq. (4). (**B** and **C**) The dependences of parameters *T*_0_ and *K*_agg_ calculated from Eq. (4) on the GAPDH concentration, respectively.

It was of interest to investigate whether the character of the dependences of parameters *T*_0_ and *K*_agg_ on the protein concentration observed for Ph*b* remains the same for other proteins. Fig. 5 shows the analysis of the initial parts of the dependences of the light scattering intensity on temperature for thermal aggregation of GAPDH. The protein concentration was varied in the interval from 0.1 to 1.5 mg/ml. As an example, the kinetics of GAPDH aggregation at the protein concentrations of 0.1, 0.75 and 1.5 mg/ml and the results of fitting the experimental data to Eq. (4) are represented in Fig. 5A. The *R* ^2^ values for approximation of the curves shown in Fig. 5A by Eq. (4) were found to be 0.881, 0.974 and 0.990 at the GAPDH concentrations of 0.1, 0.75 and 1.5 mg/ml, respectively. The calculated values of parameters *T*_0_ and *K*_agg_ are represented in Fig. 5B and C as a function of the GAPDH concentration. As in the case of Ph*b*, parameter *T*_0_ decreases monotonously with increasing the GAPDH concentration and parameter *K*_agg_ reaches a constant value at rather high protein concentrations.

**Figure 6:**
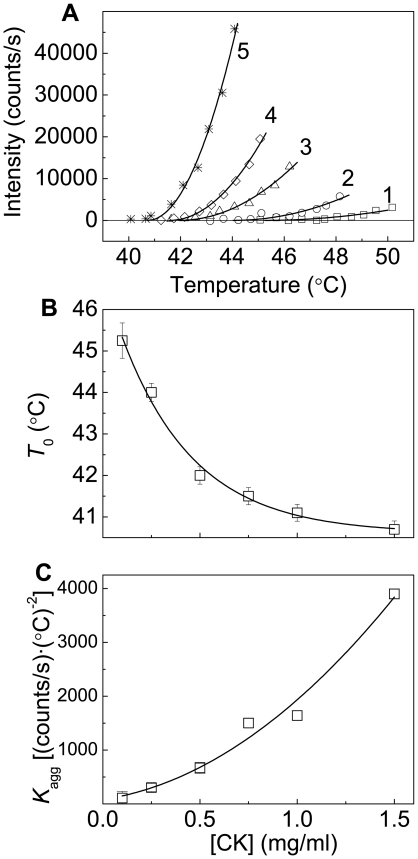
Analysis of the kinetics of CK aggregation registered in the regime of heating at the rate of 1°C/min (30 mM Hepes–NaOH buffer, pH 8.0, containing 0.1 M NaCl). (**A**) The initial parts of the dependences of the light scattering intensity on temperature obtained at the following CK concentrations: (1) 0.1, (2) 0.25, (3) 0.5, (4) 0.75 and (5) 1.5 mg/ml. Points are the experimental data. The solid curves were calculated from Eq. (4). (**B** and **C**) The dependences of parameters *T*_0_ and *K*_agg_ calculated from Eq. (4) on the CK concentration, respectively.

Fig. 6A demonstrates the applicability of Eq. (4) for the description of the initial parts of the dependences of the light scattering intensity on temperature obtained for aggregation of CK at various concentrations of the protein. The *R* ^2^ values for approximation of these dependences by Eq. (4) lie in the range from 0.846 at [CK] = 0.25 mg/ml to 0.985 at [CK] = 1.5 mg/ml. When the concentration of CK increases from 0.1 to 1.5 mg/ml, the *T*_0_ value decreases monotonously from 45.2 to 40.7°C (Fig. 6B). The value of parameter *K*_agg_ increases with increasing the CK concentration (Fig. 6C). There is no tendency for *K*_agg_ to level off at high concentrations of the protein.

**Figure 7:**
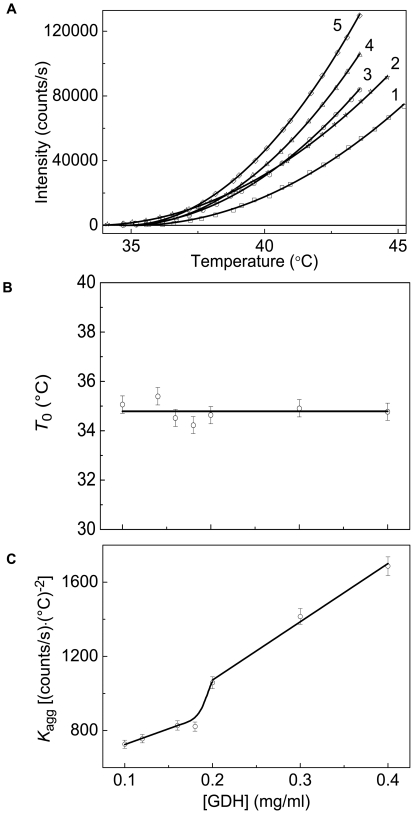
Analysis of the kinetics of GDH aggregation registered in the regime of heating at the rate of 1°C/min (0.1 M Na-phosphate buffer, pH 7.6). (**A**) The initial parts of the dependences of the light scattering intensity on temperature obtained at the following concentrations of GDH: (1) 0.1, (2) 0.12, (3) 0.2, (4) 0.3 and (5) 0.4 mg/ml. Points are the experimental data. The solid curves were calculated from Eq. (4). (**B** and **C**) The dependences of parameters *T*_0_ and *K*_agg_ calculated from Eq. (4) on the GDH concentration, respectively.

Sabbaghian et al. [11] showed that interaction of small molecules (coenzymes, allosteric effectors) with the bovine liver GDH resulted in profound changes in the extent of its thermal aggregation. It was of interest to study thermal aggregation of GDH in the regime of elevating the temperature with a constant rate. The initial parts of the dependences of the light scattering intensity on temperature obtained at various concentrations of GDH were analyzed using Eq. (4) (Fig. 7A), and the corresponding values of parameters *T*_0_ and *K*_agg_ were calculated. The *R* ^2^ values for approximation of the curves presented in Fig. 7A by Eq. (4) were found to be 0.990. The value of parameter *T*_0_ remains practically constant with variation of the GDH concentration (Fig. 7B). The average value of *T*_0_ is equal to 34.8±0.2°C. As it can be seen from Fig. 7C, parameter *K*_agg_ increases monotonously with increasing the protein concentration.

We have also analyzed the dependences of the light scattering intensity on temperature for aggregation of human interleukin 1-β presented by Chrunyk et al. [38]. These dependences were obtained at various protein concentrations. The heating rate was 0.5°C/min. The following values of parameters of Eq. (4) were found: *T*_0_ = 50.0±0.1, 48.4±0.1 and 47.5±0.1°C, *K*_agg_ = 317±15, 1130±50 and 2200±100 (°C)^−2^ for the interleukin 1-β concentration equal to 1, 2 and 4 mg/ml, respectively. The *R* ^2^ values for approximation of the experimental curves by Eq. (4) were equal to 0.965, 0.913 and 0.846 at the interleukin 1-β concentration of 1, 2 and 4 mg/ml, respectively. Thus, the enhancement of the interleukin 1-β concentration results in the diminishing of the value of parameter *T*_0_ and the increase in the *K*_agg_ value. It is worthy of mention that to characterize the temperature aggregation Chrunyk et al. [39] proposed to use a temperature corresponding to the length cut off on the temperature axis by the straight line, which is a continuation of the linear part of the dependence of the light scattering intensity on temperature reachable at the inflexion point.

In the work of Raibekas [40] the dependences of the light scattering intensity on temperature for aggregation of interleukin-1 receptor antagonist (IL-1ra) are represented in the coordinates {*I*/*I*_lim_; *T*}. The heating rate was 1°C/min. The dependences *I*/*I*_lim_ on temperature were obtained at various concentrations of IL-1ra and characterized by parameter *T*_agg_ corresponding to the level *I*/*I*_lim_ = 0.5. In the interval concentrations of IL-1ra from 1 to 10 mg/ml parameter *T*_agg_ decreases by 5.3°C, namely from 56.4 to 51.1°C (Δ*T*_agg_ = 5.3°C). We have analyzed the dependences of *I*/*I*_lim_ on temperature using an equation, which is a modification of Eq. (4):
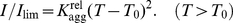
(7)In this equation 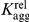 is a constant. The change in the *T*_0_ value characterizes the displacement of the dependence of *I*/*I*_lim_ on temperature along the abscissa axis, whereas parameter 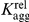 characterizes the steepness of the initial part of the dependence of *I*/*I*_lim_ on *T*. The following values of parameters of Eq. (7) were found: *T*_0_ = 53.7±0.1°C and 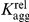 = 0.080±0.002 (°C)^−2^ (*R* ^2^ = 0.994) at [IL-1ra] = 1 mg/ml and *T*_0_ = 47.3±0.1°C and 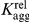 = 0.044±0.001 (°C)^−2^ (*R* ^2^ = 0.997) at [IL-1ra] = 10 mg/ml. If one assumes that the *I*_lim_ value is strictly proportional to the protein concentration, the calculations show that *K*_agg_ should increase by a factor of 5.5 when the IL-1ra concentration increases from 1 to 10 mg/ml. The displacement of the dependence of *I*/*I*_lim_ on temperature in this concentration interval was characterized by the change in parameter *T*_0_, and Δ*T*_0_ was equal to 6.4°C. This value is less than Δ*T*_agg_ (5.3°C). The discrepancy between the values of Δ*T*_0_ and Δ*T*_agg_ is explained by the lesser steepness of the initial part of the dependence of *I*/*I*_lim_ on temperature and, consequently, the lesser slope of this dependence in the inflexion point at [IL-1ra] = 10 mg/ml.

Thus, the quantitative analysis of the initial parts of the dependences of the light scattering intensity on temperature using Eq. (4) allows us to estimate the threshold temperature *T*_0_ and parameter *K*_agg_, which may be considered as a measure of the rate of aggregation. Parameter *T*_0_ has a clear physical sense. When the measurement of the light scattering intensity is used for registration of protein aggregation, the initial increment of the light scattering intensity corresponds to the instant of the formation of the start aggregates containing hundreds of denatured protein molecules [25], [26]. The value of parameter *T*_0_ is a function of the protein concentration and, as a rule, decreases with increasing the protein concentration. As it could be expected, the value of parameter *K*_agg_ characterizing the aggregation rate tends to grow as the protein concentration increases. In some cases the leveling off for the *K*_agg_ value is observed at rather high concentrations of the protein, as with Ph*b* and GAPDH. Such unexpected decrease in the growth of the aggregation rate with increasing protein concentration may have a simple explanation. When the protein concentration increases, the diminishing of the initial aggregation temperature (*T*_0_) occurs, and, consequently, the initial rate of aggregation is measured in the region of lower temperatures.

Two parameters we proposed to characterize the temperature dependence of the light scattering intensity (*T*_0_ and *K*_agg_) provide more detailed information on the character of protein aggregation than parameter *T*_agg_. Really, the value of parameter *T*_agg_ is determined by two factors, namely the threshold temperature *T*_0_ and the steepness of the initial part of the dependence of the light scattering intensity on temperature expressed by parameter *K*_agg_ (or parameter 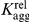).

Apart from the protein concentration there is another factor affecting the shape on the light scattering intensity versus temperature plot, namely the heating rate. One could expect that an increase in the heating rate would result in the displacement of the dependence of the light scattering intensity on temperature towards higher temperatures. Actually, such a picture was observed for aggregation of α-amylase from *Bacillus amyloliquefaciens* (BAA). Duy and Fitter [41] studied aggregation of BAA at various values of the heating rate (*v*). The protein concentration was 50 µg/ml. The experimental data were represented by the coordinates {*I*/*I*_lim_; *T*}. An analysis of the dependences of *I*/*I*_lim_ on temperature based on Eq. (7) gives the following values of parameters: *T*_0_ = 74.0±0.1°C and 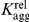 = 0.0052±0.0002 (°C)^−2^ (*R* ^2^ = 0.964) at *v* = 0.1°C/min and *T*_0_ = 79.8±0.1°C and 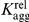 = 0.0070±0.0005 (°C)^−2^ (*R* ^2^ = 0.949) at *v* = 1.0°C/min. Thus the enhancement of the heating rate from 0.1 to 1.0°C/min results in the displacement of the dependence of the light scattering intensity on temperature along the abscissa axis by 5.8°C towards higher temperatures. The change in the steepness of the initial part of the dependence of the light scattering intensity on temperature is not marked. Parameter 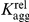 increases by a factor of 1.35, when the heating rate is changed from 0.1 to 1.0°C/min. It should be noted that the change in the value of parameter *T*_agg_ corresponding to the level *I*/*I*_lim_ = 0.5 in this interval of the heating rates was equal to 3.3°C. Consequently, Δ*T*_agg_ is less than Δ*T*_0_. Apart from the light scattering measurements the authors studied thermal unfolding of BAA using intrinsic fluorescence spectroscopy. Unfolding transitions were analyzed in terms of wavelength shifts of the emission peak and by calculating the peak ratios of intensities as measured at 330 nm and 350 nm (excitation wavelengths of 280 and 295 nm were applied). It was shown that for both heating rates (0.1 and 1.0°C/min) aggregation that was characterized by the normalized change in the light scattering intensity appeared rather concurrent with respect to the unfolding transition as obtained from tryptophan fluorescence.

### Estimation of the Size of Protein Aggregates

**Figure 8:**
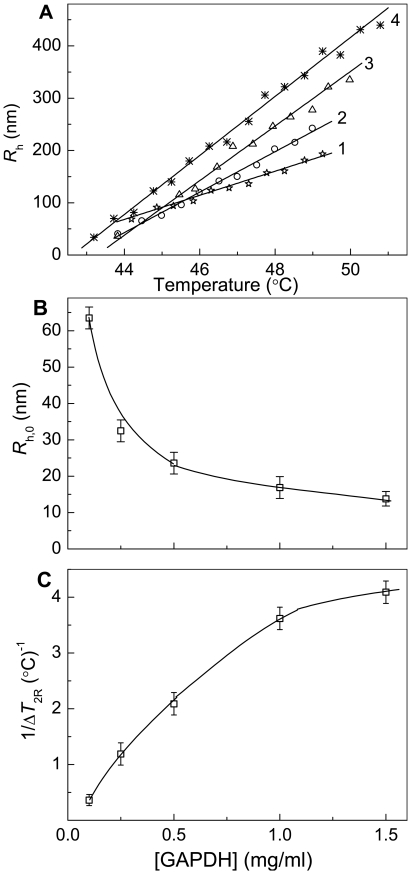
Estimation of the size of the protein aggregates formed in the course of GAPDH aggregation registered in the regime of heating at the rate of 1°C/min (10 mM Na-phosphate buffer, pH 7.5, containing 0.1 M NaCl). (**A**) The initial parts of the dependences of the hydrodynamic radius (*R*_h_) of the protein aggregates on temperature obtained at the following GAPDH concentrations: (1) 0.1, (2) 0.25, (3) 0.5 and (4) 1.5 mg/ml. (**B** and **C**) The dependences of parameter *R*_h,0_ and the reciprocal value of parameter Δ*T*_2R_ calculated from Eq. (8) on the GAPDH concentration, respectively.

Consider what additional information on the aggregation process studied in the regime of heating with a constant rate may be obtained from the measurements of the size of the protein aggregates. Fig. 8A shows the initial parts of the dependences of the hydrodynamic radius (*R*_h_) of the protein aggregates on temperature obtained for GAPDH aggregation at various concentrations of the protein. These dependences are linear, and the following equation was used for their analysis [24], [25]:
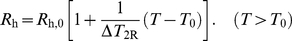
(8)In this equation *R*_h,0_ is the hydrodynamic radius of the start aggregates, *T*_0_ is the temperature at which the start aggregates appear and Δ*T*_2R_ is the temperature interval over which the hydrodynamic radius of the protein aggregates increases from *R*_h,0_ to 2*R*_h,0_. The reciprocal value of parameter Δ*T*_2R_ is a measure of the aggregation rate. The higher the 1/Δ*T*_2R_ value, the higher is the aggregation rate. Taking *T*_0_ in this equation to be identical to parameter *T*_0_ in Eq. (4), we can calculate other parameters, namely *R*_h,0_ and Δ*T*_2R_. As it can be seen from Fig. 8B, the marked decrease in the *R*_h,0_ value occurs with increasing GAPDH concentration (from 64±3 nm at [GAPDH] = 0.1 mg/ml to 14±3 nm at [GAPDH] = 1.5 mg/ml). Such a decrease in the *R*_h,0_ value is accompanied by the increase in the rate of aggregation expressed by the reciprocal value of parameter Δ*T*_2R_ (Fig. 8C).

**Figure 9:**
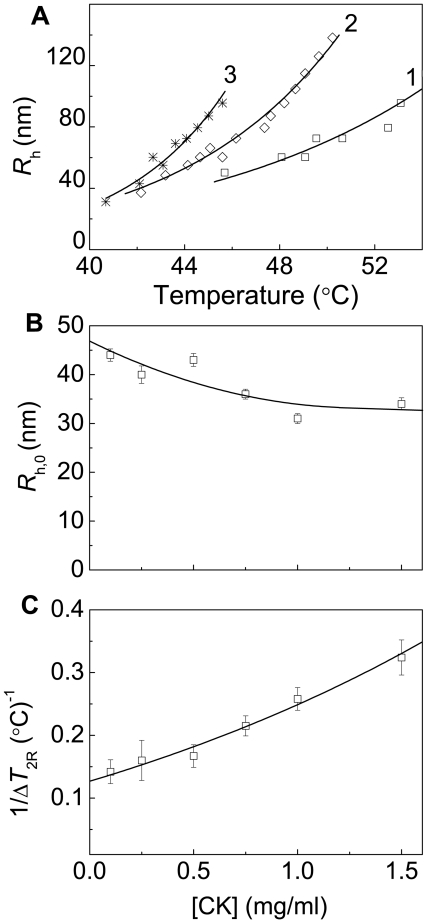
Estimation of the size of the protein aggregates formed in the course of CK aggregation registered in the regime of heating at the rate of 1°C/min (30 mM Hepes–NaOH buffer, pH 8.0, containing 0.1 M NaCl). (**A**) The initial parts of the dependences of the hydrodynamic radius (*R*_h_) of the protein aggregates on temperature obtained at the following CK concentrations: (1) 0.1, (2) 0.75 and (3) 1.5 mg/ml. (**B** and **C**) The dependences of parameter *R*_h,0_ and the reciprocal value of parameter Δ*T*_2R_ calculated from Eq. (9) on the GAPDH concentration, respectively.

Fig. 9A shows the initial parts of the dependences of the hydrodynamic radius of protein aggregates on temperature obtained for CK aggregation at various concentrations of the protein. These dependences are exponential, and the following equation was used for their analysis [24]–[26]:
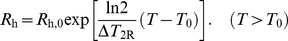
(9)In this equation Δ*T*_2R_ is the temperature interval over which the hydrodynamic radius is doubled. When the concentration of the protein increases, a slight diminishing of the *R*_h,0_ value takes place (from 44±2 nm at [CK] = 0.1 mg/ml to 34±2 nm at [CK] = 1.5 mg/ml) with simultaneous increase in the 1/Δ*T*_2R_ value (Fig. 9B and C).

**Figure 10:**
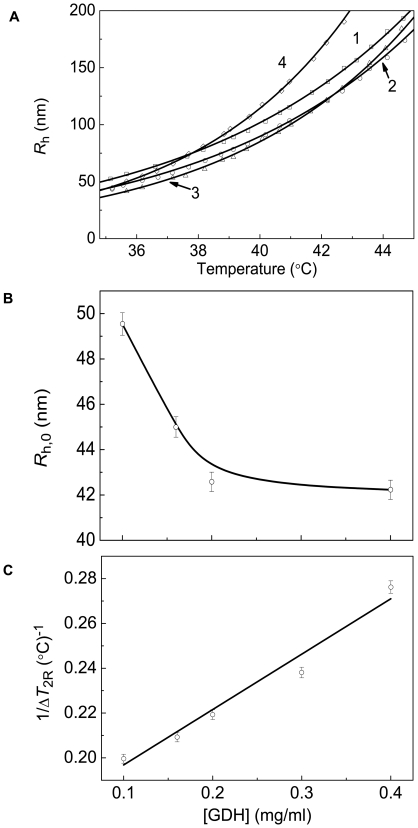
Estimation of the size of the protein aggregates formed in the course of GDH aggregation registered in the regime of heating at the rate of 1°C/min (0.1 M Na-phosphate buffer, pH 7.6). (**A**) The initial parts of the dependences of the hydrodynamic radius (*R*h) of the protein aggregates on temperature obtained at the following GDH concentrations: (1) 0.1, (2) 0.2, (3) 0.3 and (4) 0.4 mg/ml. (**B** and **C**) The dependences of parameter *R*_h,0_ and the reciprocal value of parameter Δ*T*_2R_ calculated from Eq. (9) on the GDH concentration, respectively.

As is with CK, the initial parts of the dependences of the hydrodynamic radius of protein aggregates on temperature obtained for GDH aggregation at various concentrations of the protein are exponential (Fig. 10A). The values of parameters *R*_h,0_ and 1/Δ*T*_2R_ as a function of the GDH concentration are represented in Fig. 10B and C. The increase in the GDH concentration results in the diminishing of the *R*_h,0_ value with simultaneous increase in the 1/Δ*T*_2R_ value.

The following explanation can be proposed for the decrease in parameter *T*_0_ with increasing the protein concentration (Fig. 4–6). The increase in the protein concentration favors nucleation and further formation of start aggregates. Therefore one may expect that the temperature corresponding to the moment of origination of the start aggregates will decrease with increasing the protein concentration. This picture resembles the well-known phenomenon of the decrease in the duration of the lag period on the kinetic curves of aggregation registered at a fixed temperature when the concentration of the model protein increases (see, for example [17], [26], [35], [36]).

We can interpret the linear character of the dependence of the hydrodynamic radius of protein aggregates on temperature observed for GAPDH as an indication of the fulfillment of the diffusion-limited aggregation regime, wherein the rate of aggregation is limited by diffusion of the colliding particles (the so-called regime of diffusion-limited cluster–cluster aggregation) [24], [25]. In other words, the sticking probability for the colliding particles for this regime of aggregation is equal to unity. We interpret the exponential character of the dependence of the hydrodynamic radius of the protein aggregates on temperature observed for CK and GDH as an indication for the fulfillment of the regime of reaction-limited cluster-cluster aggregation wherein the sticking probability for the colliding particles is lower than unity [24]–[26].

Consider the differences between the model of protein aggregation used in the present work and models proposed by other investigators. A general model of protein aggregation was recently elaborated by Li and Roberts [42]. This model takes into account the conformational transition of monomers between folded and unfolded states, nucleation of the smallest aggregate species, growth of aggregates via chain polymerization and aggregate growth due to aggregate-aggregate association. When studying the kinetics of thermal aggregation of proteins by dynamic light scattering, we observed that at the moment of time when the initial increment in the light scattering intensity is observed, protein aggregates containing hundreds of denatured protein molecules are registered. These primary aggregates were called the start aggregates. We could not detect intermediate states between the non-aggregated form of the protein and the start aggregates. Thus we infer that the formation of the start aggregates proceeds on the all-or-none principle. This pattern was demonstrated for thermal aggregation of β_L_-crystallin from bovine lens [12], [37], GAPDH from rabbit skeletal muscle [6], [23], [35], [37], [43], yeast alcohol dehydrogenase I [26], [37], tobacco mosaic virus coat protein [37], [44], Ph*b* from rabbit skeletal muscle [5], [23], [29], [37], [43], [45], aspartate aminotransferase from pig heart mitochondria [46] and creatine kinase from rabbit skeletal muscle [7]. On the basis of these experimental data a new model of protein aggregation was proposed [12], [23], [36], [37]. According to this model nuclei are assembled rather quickly in the start aggregates and further growth of aggregates is due to sticking of the start aggregates and aggregates of higher order. It was demonstrated that the size of the start aggregates is independent of the concentration of the protein involved in aggregation. This fact allowed us to draw an analogy between the formation of the start aggregates and micelle formation. In the latter case the micelles of a definite size are formed when the critical micelle concentration is achieved. Such an analogy offers an explanation of why the formation of start aggregates proceeds according to the all-or-none principle.

The idea on the formation of primary aggregates in the course of protein aggregation was also used by Durand and coworkers.When studying thermal aggregation of β-lactoglobulin at pH 7.0 by size exclusion chromatography, Durand et al. [47]–[51] showed that there was a clear separation between a narrow peak that corresponded to residual native protein and a broad peak that corresponded to the aggregates. This observation implied that the aggregates of the minimum size contained many monomers and that no or very few stable oligomers were formed. The authors supposed that the first stage of thermal aggregation of β-lactoglobulin was the step of the formation of “primary aggregates”.

Shiraki and coworkers [52] studied heat-induced aggregation of lysozyme at around the isoelectric point. Dynamic light scattering and transmission electron microscopy revealed that aggregation occurred in a two-step process: formation of start aggregates, followed by further growth mediated by their sticking with diffusion-limited cluster-cluster aggregation.

Recent investigations of dithiothreitol-induced aggregation of alpha-lactalbumin and insulin using dynamic light scattering convincingly demonstrate that the aggregation process proceeds through the stage of formation of the start aggregates [53], [54].

It is notable that according to present views the assembly of amyloid fibrils proceeds through the stage of the formation of protofibrils. Association of protofibrils results in the formation of fibrils and then clusters of fibrils and bundles [55], [56]. It is evident that protofibrils are equivalent to the start aggregates in the case of aggregation producing amorphous aggregates (this analogy was discussed in Golub et al. [23]).

### Screening of the Ligands Affecting Denaturation or Aggregation of Ph*b* and GDH on the Basis of the Data of the Aggregation Kinetics

**Figure 11:**
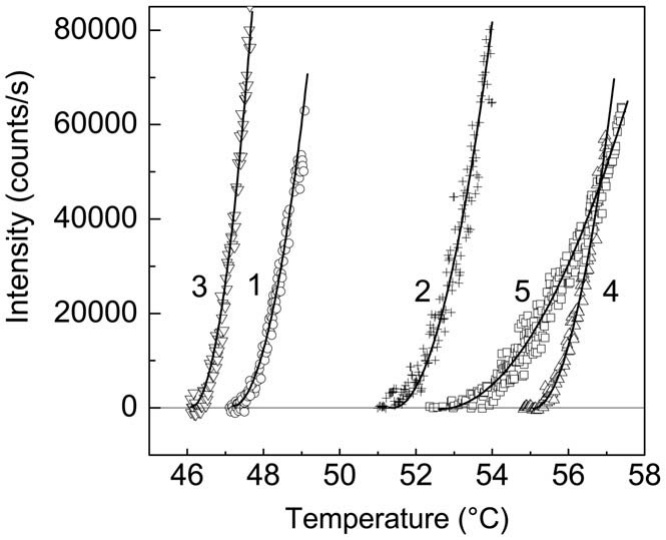
Effect of different agents on Ph*b* aggregation. The initial parts of the dependences of the light scattering intensity on temperature for aggregation of Ph*b* (0.5 mg/ml) in the presence of the following agents: (1) control, (2) 1 mM AMP, (3) 0.19 M HP-β-CD, (4) 1 M TMAO and (5) α-crystallin at the concentration of 1 mg/ml. Points are the experimental data. The solid curves were calculated from Eq. (4).

Consider the use of this new approach to characterization of the aggregation rate for the quantitative estimation of the ability of various agents to suppress protein aggregation. Our investigations have shown that Ph*b* is a convenient model for testing the compounds affecting protein denaturation and aggregation [4], [5], [25], [29], [45], [57]–[60]. Fig. 11 shows the effect of some agents on Ph*b* aggregation. The suppression of aggregation in the presence of 1 mM AMP is accompanied by the increase in the *T*_0_ value from 47.2°C (curve 1; the control) to 51.4°C (curve 2; 1 mM AMP) and the decrease in parameter *K*_agg_ from (1.83±0.02)⋅10^5^ (curve 1) to (1.20±0.02)⋅10^5^ (counts/s)⋅(°C)^−2^ (curve 2). Based on the data on kinetics of thermal inactivation of Ph*b* and DSC data [58], [60], we can conclude that the anti-aggregation ability of allosteric activator AMP is due to the stabilization of the native Ph*b* molecule. The acceleration of Ph*b* aggregation in the presence of HP-β-CD (curve 3 in Fig. 11) is accompanied by the decrease in the *T*_0_ value to 46.1°C and the increase in parameter *K*_agg_ to (3.27±0.03)⋅10^5^ (counts/s)⋅(°C)^−2^. This action of HP-β-CD is caused by the destabilization of the Ph*b* molecule as a result of binding of HP-β-CD to the native form of the enzyme and early intermediates of Ph*b* unfolding as suggested by the data on the kinetics of thermal inactivation and DSC data [45]. It is known that osmolytes stabilize the protein structure [61]–[63]. Therefore, when testing the effect of osmolytes on protein aggregation, one can expect that osmolytes will reveal the protective action. Fig. 11 demonstrates that a markedly expressed retardation of thermal aggregation of Ph*b* is observed in the presence of 1 M TMAO. The *T*_0_ value increases to 55.1°C and the *K*_agg_ value decreases to (1.54±0.02)⋅10^5^ (counts/s)⋅(°C)^−2^ (curve 4). At last, it is of interest to discuss the effect of the agents of protein nature responsible for suppression of protein aggregation in the cell, namely small heat shock proteins. Meremyanin et al. [25], [29] showed that α-crystallin, one of the representatives of small heat shock protein family, effectively hindered thermal aggregation of Ph*b*. Curve 5 in Fig. 11 presents an initial part of the dependence of the light scattering intensity on temperature for Ph*b* aggregation measured in the presence of α-crystallin at the concentration of 1 mg/ml. The protective action of α-crystallin is reflected in the increase in the *T*_0_ value (to 52.6°C) and the marked decrease in the *K*_agg_ value (to (0.27±0.01)⋅10^5^ (counts/s)⋅(°C)^−2^). The *R* ^2^ values for approximation of the curves 1–5 shown in Fig. 11 by Eq. (4) were found to be 0.981, 0.954, 0.987, 0.986 and 0.972, respectively. The values of parameters of *T*_0_ and *K*_agg_ obtained during testing of the effect of various agents on Ph*b* aggregation are given in Table 1.

**Table 1:** Parameters of aggregation of Ph*b* (0.5 mg/ml) in the presence of different ligands (0.08 M Hepes-buffer, pH 6.8, containing 0.1 M NaCl).

Ligand	*T*_0_ (°C)	*K*_agg_ [(counts/s)⋅(°C)^−2^]
-	47.2±0.1	(1.83±0.02)⋅10^5^
AMP (1 mM)	51.4±0.1	(1.20±0.02)⋅10^5^
HP-β-CD (19 mM)	46.1±0.1	(3.27±0.03)⋅10^5^
TMAO (1 M)	55.1±0.1	(1.54±0.02)⋅10^5^
α-crystallin (1 mg/ml)	52.6±0.1	(0.27±0.01)⋅10^5^

**Figure 12:**
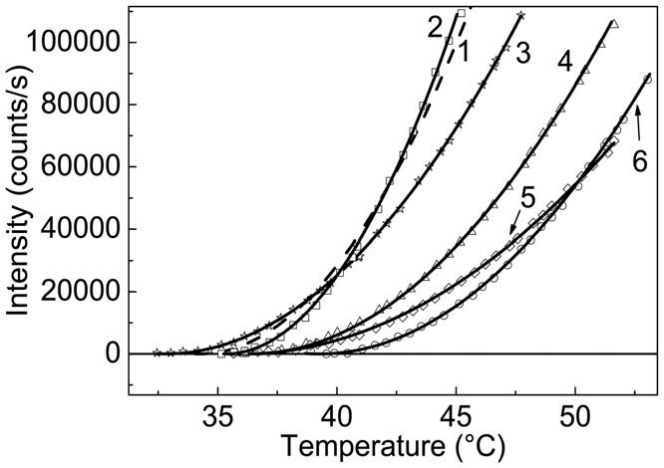
Effect of different agents on GDH aggregation. The initial parts of the dependences of the light scattering intensity on temperature for aggregation of GDH (0.12 mg/ml) in the presence of the following agents: (1) control; (2) 0.2 mM NADH; (3) 50 mM L-glutamate; (4) 0.2 mM NADH+0.5 mM ADP; (5) 50 mM L-leucine and (6) 0.5 mM ADP. Points are the experimental data. The solid curves were calculated from Eq. (4).

Fig. 12 shows the effect of specific ligands (NADH, ADP, L-glutamate and L-leucine) on aggregation of GDH studied in the regime of heating with a constant rate. The values of parameters *T*_0_ and *K*_agg_ are given in Table 2. The increase in parameter *K*_agg_ in the presence of 0.2 mM NADH means that NADH displays the destabilizing action on the enzyme. Judging from the *K*_agg_ values, the enhancement of GDH stability is observed in the presence of allosteric activators, ADP and L-leucine, and in the presence of substrate, L-glutamate. ADP (0.5 mM) eliminates the destabilizing effect of NADH (0.2 mM). NAD has no effect on GDH aggregation (see Table 2).

**Table 2:** Parameters of aggregation of GDH (0.12 mg/ml) in the presence of different ligands (0.1 M Na-phosphate buffer, pH 7.6).

Ligand	*T*_0_ (°C)	*K*_agg_ [(counts/s)⋅(°C)^−2^]
-	34.3±0.1	(8.7±0.1)⋅10^2^
NADH (0.2 mM)	35.0±0.1	(11.6±0.2)⋅10^2^
NAD (0.2 mM)	34.5±0.1	(8.4±0.1)⋅10^2^
ADP (0.5 mM)	39.3±0.1	(4.7±0.1)⋅10^2^
NADH (0.2 mM)+ADP(0.5 mM)	36.4±0.1	(4.7±0.1)⋅10^2^
L-Leucine (50 mM)	36.0±0.1	(2.8±0.1)⋅10^2^
L-Glutamate (50 mM)	32.8±0.1	(4.9±0.1)⋅10^2^

**Figure 13:**
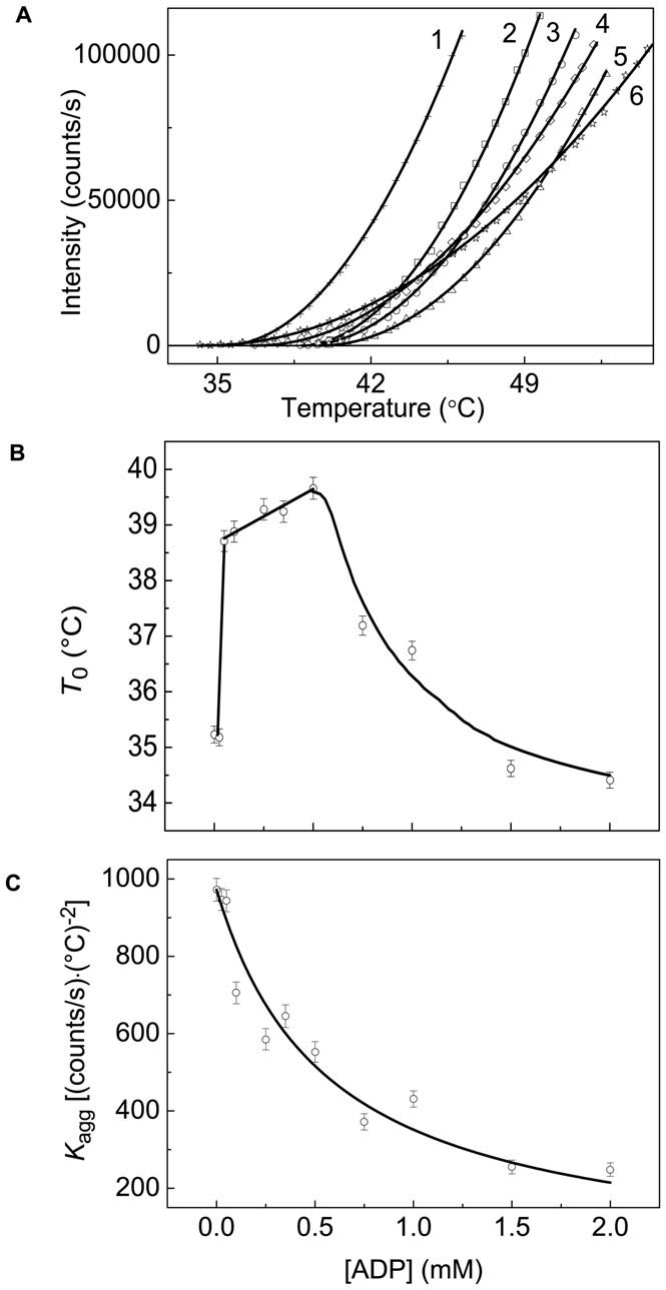
Effect of ADP on GDH aggregation. (**A**) The initial parts of the dependences of the light scattering intensity on temperature for aggregation of GDH (0.16 mg/ml) in the presence of the following concentrations of ADP: (1) 0, (2) 0.05, (3) 0.1, (4) 0.5 and (6) 2 mM. (**B** and **C**) The dependences of parameters *T*_0_ and *K*_agg_ calculated from Eq. (4) on the ADP concentration, respectively. The solid curve in panel **C** was calculated from Eq. (10) at *K*_diss_ = 0.52 mM.

The effect of ADP on aggregation of GDH was studied in greater detail. Fig. 13A shows the initial parts of the dependences of the light scattering intensity on temperature obtained for GDH aggregation at various concentrations of ADP. The values of parameters *T*_0_ and *K*_agg_ as a function of the ADP concentration are represented in Fig. 13B and C. A sharp increase in parameter *T*_0_ is observed in the presence of 0.05 mM ADP, namely from 35.2°C at [ADP] = 0 to 38.7°C at [ADP] = 0.05 mM (Fig. 13B). The changes in parameter *T*_0_ in the interval of ADP concentration from 0.05 to 0.5 mM are insignificant. The average value of *T*_0_ in this interval of ADP concentrations is equal to 39.1±0.2°C. A decrease in parameter *T*_0_ is observed at the ADP concentrations higher than 0.5 mM. As it follows from Fig. 13C, the increase in ADP concentration is accompanied by the diminishing of parameter *K*_agg_. The dependence of *K*_agg_ on ADP concentration in the interval 0.05–0.5 mM was analyzed using the following equation:
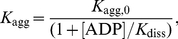
(10)where *K*_agg,0_ is the value of *K*_agg_ at [ADP] = 0, and *K*_diss_ is the microscopic dissociation constant for the ADP-GDH complex. The *K*_diss_ value was found to be 0.52±0.07 mM. It is reasonable to suppose that this value of *K*_diss_ corresponds to the temperature of 39.1°C.

### Conclusion

This article has described a new approach to testing the agents affecting the stability of proteins. The approach is based on the quantitative analysis of the initial increment in the light scattering intensity caused by aggregation of the denatured protein molecules in the experiments where aggregation is studied in the regime of the elevation of temperature with a constant rate. The proposed approach allows estimating the temperature at which the initial increase in the light scattering intensity is registered (*T*_0_) and establishing parameter *K*_agg_, which characterizes the rate of aggregation. Temperature *T*_0_ is a moment of the origination of the start aggregates. Such aggregation systems may be used for testing the compounds affecting protein stability as a result of direct binding to the native protein molecule (for example, substrates and modifiers of the enzymes); the compounds stabilizing the protein structure owing to the effects of excluded volume (for example, osmolytes); and the compounds possessing chaperone-like activity (for example, small heat shock proteins). If we want to select agents affecting exclusively the stage of denaturation, we should demonstrate that these agents have no effect on the stage of aggregation. In turn, this would require additional aggregation systems where a preliminary denatured protein undergoes thermal aggregation. A protein denatured by ultraviolet radiation may be used for this purpose [64]. Test-systems based on aggregation of UV-irradiated proteins allow registering the direct action of the agents under study on the stages of aggregation.

When discussing the practical utility of the method of study of protein-ligand interactions proposed in the present work, we should like to note, first of all, that this method may be used for characterization of the proteins themselves. The results of investigation of protein stability in buffer solutions of different composition may be useful for selection of optimal buffer for purification procedure and storage of the proteins under study. The protein mutant forms obtained by genetic engineering may also be characterized by the method under discussion. Furthermore, screening against cofactors, substrate analogs, effectors and metals may be used to establish the conditions that favor protein stability. These stabilizing agents may be used during protein crystallization to improve the crystallization rate. Finally, an important line of application of the screening procedure based on the measurements of the light scattering intensity increase, which accompanies protein aggregation, is the search for compounds of potential medicinal significance.
